# The Best Evidence for the Prevention and Management of Lower Extremity Deep Venous Thrombosis After Gynecological Malignant Tumor Surgery: A Systematic Review and Network Meta-Analysis

**DOI:** 10.3389/fsurg.2022.841275

**Published:** 2022-03-22

**Authors:** Jiaqi Hu, Yidan Geng, Jingyi Ma, Xuefan Dong, Shuqin Fang, Jianli Tian

**Affiliations:** ^1^College of Nursing, Chengde Medical University, Chengde, China; ^2^Department of Gynecology, Affiliated Hospital of Chengde Medical University, Chengde, China

**Keywords:** deep venous thrombosis, after tumor operation, gynecological malignant, prevention, meta-analysis

## Abstract

**Background:**

To search and obtain the relevant evidence of prevention and management of lower extremity deep venous thrombosis (DVT) after gynecological malignant tumor operation and to summarize the relevant evidence.

**Methods:**

We searched the JBI evidence summary, up to date, the national comprehensive cancer network of the United States, the guide library of the National Institute of clinical medicine of the United Kingdom, PubMed, the Chinese biomedical literature database, CNKI, Wanfang, and other relevant evidence on the prevention and management of DVT in patients with gynecological malignant tumors. It includes clinical practice guidelines, best practice information book, expert consensus, evidence summary, original research, etc. The retrieval time limit is from database establishment till August 20, 2021. Two researchers independently evaluated the literature quality, combined with professional judgment, and extracted the literature that met the standards.

**Results:**

Finally, 18 literatures were included, including eight guidelines, three evidence summaries, four systematic evaluations, two expert consensuses, and one best practice information volume. A total of 26 pieces of the best evidence on the prevention and management of postoperative venous thrombosis in gynecological malignant tumors were summarized. It includes risk assessment, drug prevention, mechanical prevention, management strategy, and health education.

**Conclusion:**

This study summarized the best evidence of risk, prevention, and health management of DVT in postoperative patients with gynecological malignant tumors to provide evidence-based basis for clinical nurses and to improve the nursing level.

## Introduction

According to the latest data released by the international agency for research on cancer (IARC) (1), the global cancer burden has risen to 19.3 million new cases and 10 million deaths in 2020. The second leading cause of death in cancer patients is deep venous thromboembolism (DVT) (2). The incidence of postoperative DVT in gynecological malignant tumors is as high as 19.6–38% (3). DVT refers to abnormal coagulation of blood in the deep vein, which blocks venous lumen and leads to abnormal venous reflux (4). If it is not found and effectively controlled in time, it can cause pulmonary embolism and even disability and death (5). Therefore, the importance of prevention and management of postoperative deep venous thrombosis (DVT) in gynecological malignant tumors cannot be ignored. There are many studies on DVT prevention at home and abroad, mainly in the form of literature review, and the research method is relatively single. Although China has published guidelines for the prevention of thrombotic diseases, there is a lack of guidelines or best practice manuals for the prevention and management of postoperative DVT of gynecological malignant tumors. This study systematically searched the relevant literature on the prevention and management of postoperative DVT of gynecological malignant tumors at home and abroad, evaluated, classified, and summarized the evidence by using the method of evidence-based nursing, and finally formed the best evidence to provide references for the prevention and management of postoperative DVT of gynecological malignant tumors.

## Materials and Methods

### Search Strategy and Selection Criteria

The problem development tool of evidence-based nursing center of Fudan University was adopted to form evidence-based nursing problems based on pipost (6). They included the following: (1) evidence application target population (P): patients with gynecological malignant tumors after operation; (2) intervention (I): evaluation, prevention, drug and physical intervention methods of DVT; (3) professional (P): clinicians and nurses; (4) outcome (o): incidence of DVT, patients' cognition of DVT prevention, and compliance with prevention and management measures. Acceptance, cognition, and implementation of evidence by medical staff; (5) setting (s): gynecological ward and operating room; (6) type of evidence (T): guide, expert consensus, best practice information book, system evaluation, and evidence summary.

The evidence retrieval is carried out according to the “6S” evidence model (7). The databases searched are: computer search up to date (Chinese version), JBI evidence summary, National Comprehensive Cancer Network (NCCN), National Institute for health and care excellence (NICE), PubMed, Chinese biomedical literature database, CNKI, Wanfang. The English search subject words are: “deep vein thrombosis/deep venous thrombosis/venous thrombosis/deep venous thrombosis/thrombemembolism/” cancer/ gynecological cancers/gynecological malignant tumors “risk assessment/assessment/risk factors.”

Inclusion criteria are as follows: the subjects were patients after radical operation of gynecological malignant tumors, and the evidence applicable to patients after operation of gynecological malignant tumors were also included; research involving evaluation and intervention of DVT; outcome measures included complications caused by DVT; research types include evidence summary, best clinical practice information book, guidelines (2016–2021), expert consensus, systematic evaluation, etc. The research language is English. Exclusion criteria include the following: incomplete literature and information that cannot obtain the full text, where the literature includes abstract, plan, report, and draft; and studies that did not pass literature evaluation.

### Data Collection

Two researchers with an evidence-based medicine background independently completed the quality evaluation of the included literature to determine the inclusion and exclusion criteria of the literature. In case of disagreement, the evidence-based nursing team composed of nursing management and gynecological nursing experts in our hospital shall decide to include or eliminate the research. After the evidence was extracted, another researcher carefully checked it again to ensure the accuracy of the data. When the evidence conclusions from different sources conflict, the inclusion principle followed in this study is evidence-based evidence first, high-quality evidence first, and the latest published guidelines and authoritative literature first (8).

Literature quality evaluation criteria: (1) the guideline adopts the British clinical guidelines research and evaluation system (9) (the approval of guidelines for research and evaluation instrument, agree II) updated in 2012, including 6 independent fields and 23 items. Grade seven scoring method is adopted (one means highly disagree and seven means highly agree). The score of each field is the sum of the scores of each item in the field and is normalized to the percentage of the highest possible score in the field. Standardized percentage of each field = (actual score–minimum possible score)/(maximum possible score–minimum possible score) ×100%. The higher the score, the higher the quality of the guide. It is divided into three recommendation levels: Grade A, and 6 areas with scores ≥ 60%, which is strongly recommended; Grade B, with scores of 30–60% in at least three fields, which is recommended; Grade C, with scores ≤ 30 in at least three fields, which is not recommended temporarily (9). (2) Expert consensus, systematic evaluation, case-control, and randomized control were used to evaluate the literature according to the evaluation criteria of JBI evidence-based health care center in Australia (2016) (10). The evaluation items of the evaluation tools of different research types are different, but the unified evaluation criteria adopted by each item are “yes, no and unclear”; (3) the quality evaluation of evidence summary is directly based on the original research literature to classify the evidence level and the recommendation level, and the quality evaluation tools for various original studies of the JBI evidence-based health care center in Australia are used for evaluation (11); (4) the quality evaluation of clinical decision-making and best practice can be traced back to the original literature on which the evidence is based, and the relevant evaluation criteria are selected for evaluation according to the literature type.

### Statistical Analysis

A network meta-analysis was conducted on the occurrence of lower extremity deep venous embolism in the perioperative period of gynecological malignant tumors. We use an extension of the frequency random-effects model for multiple comparisons. The network meta-analysis was performed using Stata's mvmeta command. We used the raw data in the 2 × 2 table in the analysis. The odds ratio, risk ratio or ratio, and appropriate variance are calculated and combined in the analysis to obtain the overall relative risk. An interaction term is added to the model to estimate the difference between direct and indirect evidence results. All potential interactions were tested in an overall test to determine whether there were any inconsistencies in our network meta-analysis. The following sensitivity analyses are planned: each study design, each funding source (whether industry-sponsored or not), within the first user, and based on the risk of bias. All statistical analyses were performed using Stata version 22.0.

## Results

### Characteristics of Included Studies

**Figure 1:**
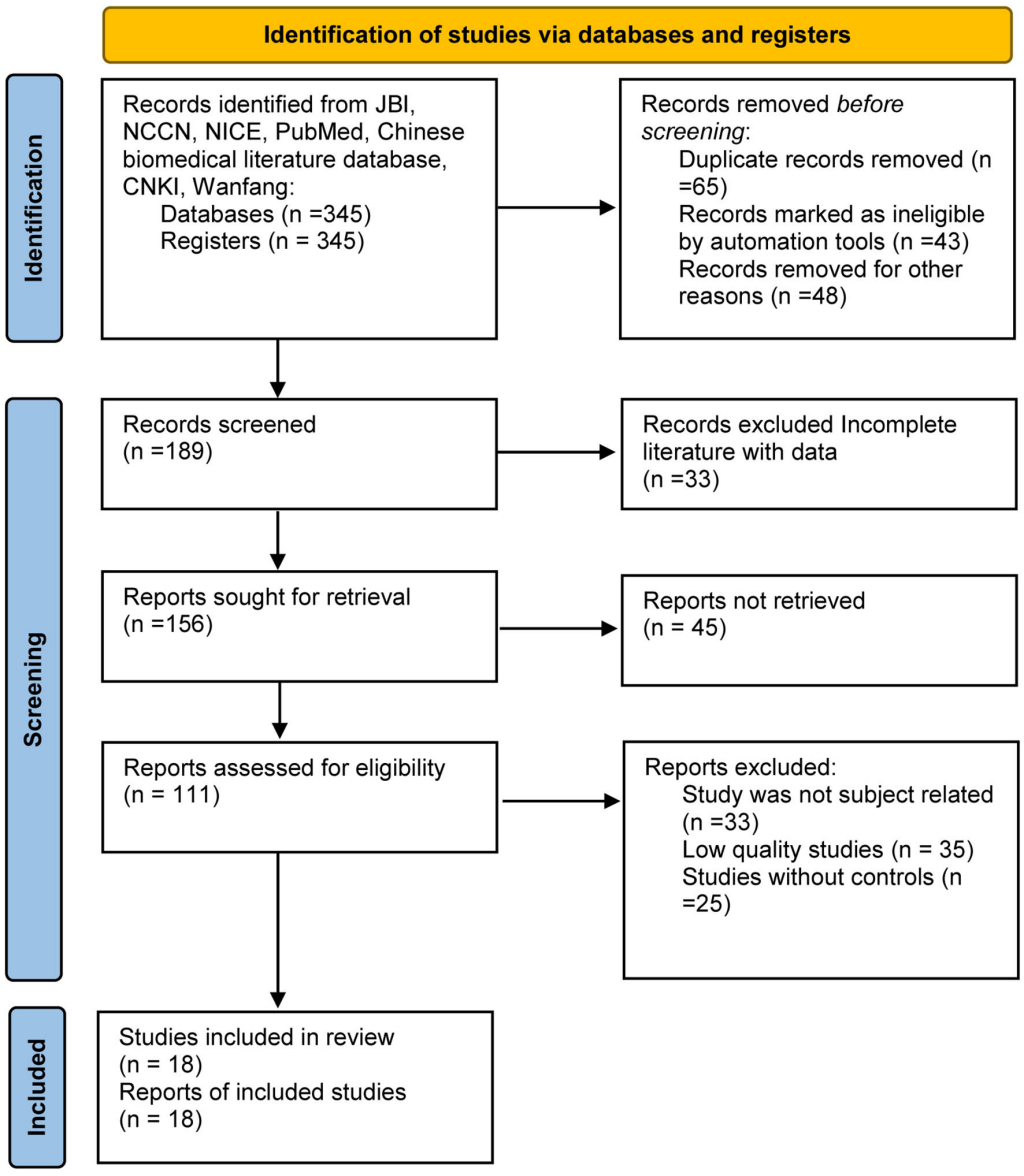
PRISMA flow diagram for the meta-analysis.

A total of 345 relevant literatures were initially retrieved and 18 were finally included, including eight guidelines, three evidence summaries, four systematic evaluations, two expert consensuses, and 1 best practice information volume. The PRISMA flow diagram is shown in Figure 1. The relevant information of the included literature is shown in Table 1 (1, 6, 12–29). A total of 8 guidelines (12–18, 30) were included in this study, including two from the national comprehensive cancer network of the United States, two from the guide library of the National Institute of clinical medicine of the United Kingdom, two from PubMed, and two from HowNet. The quality evaluation results of the guidelines are shown in Table 2 (1, 3, 5, 6, 12–15).

**Table 1:** General information included in the literature.

**Number**	**Included literature**	**Research topics**	**Nature of evidence**	**Database source**	**Publication time**
1	NCCN (1)	GLOBOCAN Estimates of Incidence	Guide	NCCN	2021
2	Farge et al. (12)	Treatment and prophylaxis of venous thromboembolism	System evaluation	Pubmed	2019
3	Liew et al. (13)	Asian venous thromboembolism guidelines	System evaluation	Pubmed	2017
4	Wade et al. (14)	Graduated compression stockings for the prevention of deep-vein thrombosis	System evaluation	JBI	2015
5	NICE (15)	Reducing the risk of hospital-acquired deep vein thrombosis	Guide	NICE	2019
6	Streiff et al. (16)	Cancer-associated venous thromboembolic disease	System evaluation	Pubmed	2018
7	Wu and Cheng (17)	Analysis of perioperative risk factors	System evaluation	Pubmed	2020
8	Guo et al. (18)	Coagulation alteration and deep vein thrombosis	System evaluation	Medline	2018
9	Insin et al. (19)	Prevention of venous thromboembolism	System evaluation	Cochrane Library	2021
10	Hajibandeh et al. (20)	Prevention of venous thromboembolism	System evaluation	Pubmed	2017
11	Pavon et al. (21)	Effectiveness of intermittent pneumatic compression devices	System evaluation	Cochrane Library	2016
12	Buesing et al. (22)	Deep venous thrombosis and venous thromboembolism prophylaxis	System evaluation	MedLine	2015
13	Lieberman (23)	Deep vein thrombosis prophylaxis	System evaluation	JBI	2018
14	Fan et al. (24)	Perioperative prevalence of deep vein thrombosis	System evaluation	JBI	2020
15	Li et al. (25)	Incidence and locations of deep venous thrombosis	System evaluation	Pubmed	2020
16	Matsusaki et al. (26)	Central venous thrombosis and perioperative vascular access	System evaluation	Cochrane Library	2012
17	NCCN (6)	Deep vein thrombosis and serum D-dimer	Guide	NCCN	2020
18	Gantz et al. (27)	Incidence and cost in emergency general surgery	System evaluation	Pubmed	2020

**Table 2:** Quality evaluation results included in the guidelines.

**Inclusion**	**Scope and**		**Rigor of**	**Clarity and**		**Editorial**	**Number of**	**≥30%**	**Recommendation**
**guidelines**	**purpose**	**Participants**	**production**	**clarity**	**Applicability**	**independence**	**fields ≥60% (PCs.)**	**fields**	**level**
NCCN (1)	98.32	88.74	90.43	93.34	91.65	100	6	6	A
Farge et al. (12)	100	89.67	100	98.78	97.91	100	6	6	A
Liew et al. (13)	87.51	56.38	86.28	78.41	86.52	48.82	4	6	B
Wade et al. (14)	97.22	88.89	58.31	67.87	42.16	68.52	3	6	B
NICE (15)	78.48	89.64	46.78	84.23	58.33	43.15	3	6	B
NCCN (6)	76.91	61.91	68.38	77.62	73.72	89.23	6	6	A
Nicklas et al. (3)	87.81	58.72	95.42	74.12	82.47	88.48	5	6	B
Aufwerber et al. (5)	93.32	78.72	87.28	67.43	73.34	56.52	5	6	B

Four systematic reviews (19–21, 28) were included in this study, including one (19) from PubMed, two (20, 28) from the Cochrane Library, and one (21) from MEDLINE. Among the systematic evaluation quality evaluation results by Insin et al. (19), only the evaluation result of item seven “whether the proposed further research direction is appropriate” is “unclear,” and the evaluation results of other items are “yes”. In the systematic evaluation quality evaluation results by Hajibandeh et al. (20), only the evaluation result of Item nine “whether to evaluate the possibility of publication bias” is “no,” and other items are “yes” (Kahn et al. and Pavon et al.). Only in Item four “is the retrieved database or resources sufficient?” the evaluation result is “no,” and the evaluation result of other items is “yes.” The overall quality evaluation is high. Two expert consensuses articles (1, 25) were included in this study and derived from HowNet (1) and Wanfang medical database (25), and their quality was evaluated according to the standards of the JBI evidence-based practice center in Australia. All the item options of the two items were “yes.” One best practice manual from JBI and three evidence summaries from JBI are traced back to the original literature, including one guide and two systematic reviews. One guideline (13) coincides with the literature included in this study, and the evaluation result of item four of the remaining systematic evaluation is “no” and the rest is “yes”; one item nine evaluation result of the article is “no,” and the rest is “yes”.

By summarizing the evidence of prevention and management of venous thromboembolism after gynecological malignant tumor operation, 26 pieces of evidence were formed from five aspects: risk assessment, drug prevention, mechanical prevention, management strategy, and health education. In this study, the evidence classification and evidence recommendation level system of JBI evidence-based health care center in Australia (2014) (26) was used to divide the evidence level included in this study into five levels 1–5. According to the preciseness and scientificity of the research design, the recommendation level was divided into A-level recommendation (strong recommendation) and B-level recommendation (weak recommendation).

### Risk Assessment

Evidence 1–3 summarizes the methods, contents, and high-risk factors of postoperative DVT evaluation of gynecological malignant tumors. The commonly used VTE risk evaluation tool caprini (1) scoring scale is used to calculate the total score through risk factors and assignment. The risk is divided into low risk (0–1), medium risk (2), high risk (3–4), and very high risk (≥5). Caprini thrombus risk assessment scale is used to assess high-risk groups, and its sensitivity and specificity should be improved. At present, most risk assessment tools are based on retrospective analysis, and a large sample prospective research is needed in the future.

Evidence 4–6 summarizes the evaluation methods of DVT, including imaging evaluation, test evaluation, and clinical performance evaluation. The typical clinical manifestations of DVT are swelling and pain of the affected limb, and then changes in limb skin color and temperature (not all patients have the above symptoms). The preferred imaging method for the preliminary diagnosis of DVT is Doppler ultrasound. The routine laboratory examination indexes are D-dimer and protein C/s activity. If the patient has no clinical manifestations related to DVT and if the D-dimer test is negative, unstable, or active DVT can be excluded (22). Mastering the methods and contents of evaluation is of great significance to effectively prevent and manage the occurrence of postoperative DVT of gynecological malignant tumors.

Evidence 7–10 summarizes the methods of DVT drug prevention for gynecological malignant tumors. Drug prevention should be considered when the risk of thrombosis is greater than the risk of bleeding (17). About 2–12 h before operation, the highest dose of low molecular weight heparin (13) was used to prevent postoperative venous thromboembolism in patients with gynecological malignant cancer without relevant treatment contraindications (12).

The use of low molecular weight heparin for gynecologic malignancies after laparotomy and laparoscopy was extended to 4 weeks (for patients with a high risk of venous thromboembolism and a low risk of bleeding) (13). The high incidence period of DVT is the first 6 months after cancer diagnosis. Once patients with gynecological malignant tumors are diagnosed with DVT, anticoagulant therapy should be started immediately (25). Therefore, early assessment and detection of risk factors of DVT and timely adoption of effective drug prevention can effectively reduce the incidence of DVT.

Evidence 11–15 summarizes the methods of mechanical prevention of DVT in gynecological malignant tumors, including intermittent pneumatic compression (IPC), plantar venous pump (VFP), neuromuscular electrical stimulation (20), and graded compression stockings (GCS). If there is no contraindication, it is recommended that the patient can use the portable intermittent pneumatic compression (IPC) on both legs until the end of the operation. IPC is preferred to patients with high bleeding risk. GCS is preferred and worn all day. Different models are selected according to the diameter of lower limbs, and grade I pressure is preferred. During the GCS period, the temperature, blood supply, dorsalis pedis artery pulsation, limb sensation, and leg circumference of patients' lower limb skin were evaluated every day, and the integrity and flatness of GCs were regularly checked to ensure the effectiveness of pressure (1). Inferior vena cava (IVC) can be prevented in patients with acute proximal lower extremity DVT who are absolutely contraindicated by anticoagulant treatment. The temporary or recyclable filter is preferred, and the filter shall be taken out in time after the risk of PE is relieved (13). The use of inferior vena cava filters is not recommended for routine prevention (29).

Although the latest version of the guidelines for the diagnosis and treatment of DVT (Third edition) in China affirms the role of mechanical and drug prevention, according to the investigation on the implementation status of DVT risk assessment and prevention in general surgery conducted by Shanghai DVT prevention and control alliance in 2019, the risk assessment rate of DVT in general surgery is only 61.9%, It can be reflected from the side that the risk assessment and prevention implementation of DVT in China need to be further improved (27).

Evidence 16 indicates that when patients with malignant tumors are diagnosed with DVT, the initial treatment is to use low molecular weight heparin (LMWH) (24) when the creatinine clearance rate is ≥30 ml/min. When treating venous thromboembolism in cancer patients, low molecular weight heparin or direct oral anticoagulant drugs should be used for at least 6 months (28).

Evidence 18–19 summarize the need to manage the risk of bleeding when deciding to use anticoagulants for thromboprophylaxis. When the patient's condition worsens, different anticoagulant drugs are replaced, and surgery is performed, the bleeding risk needs to be reassessed and corresponding management measures should be taken (19). Patients with gynecological malignant tumor DVT should receive anticoagulant therapy for at least 3–6 months, and patients with DVT combined with PE should receive anticoagulant therapy for at least 6–12 months. For patients with persistent DVT risk factors, indefinite anticoagulation should be considered (16).

Evidence 17 summarizes the bleeding management. When the patient has bleeding during anticoagulation, it is necessary to immediately ask the time of the last use of anticoagulant drugs, test the blood creatinine clearance and hemoglobin to quickly evaluate the coagulation function, and take corresponding treatment measures according to the severity of bleeding and the individual situation of the patient (18).

Evidence 20–21 summarize the health education on DVT prevention and management of gynecological malignant tumors, explain the relevant knowledge of thrombosis prevention to patients, and guide patients to develop scientific and reasonable eating habits and healthy lifestyle, such as quitting smoking and alcohol, controlling blood glucose and blood lipid (14). These evidence encourage patients to move and get out of bed early after operation, guide patients to exercise ankle pump to promote lower limb blood circulation, and remind patients to pay attention to the use of bed bars to prevent falling off the bed (19).

Evidence 22–23 summarizes the appropriate rehydration of postoperative patients, educating patients to drink 1,500–2,500 ml/day to avoid blood concentration (14). The upper limb with PICC catheterization side can relax and clench to promote the blood circulation of the upper limb (13).

Evidence 24–26 summarize the health education related to mechanical prevention, inform patients and family members of the risks and consequences of DVT and the necessity of taking mechanical preventive measures, and guide the correct application of mechanical pre-treatment methods, precautions, possible adverse reactions, and response plans. If GCS is used, it is recommended to wear it both during the day and at night. GCS has to be taken off every day for limb evaluation, such as skin cleanliness, temperature, dorsalis pedis artery pulsation, limb sensation, and leg circumference (23). The flatness and integrity of GCs surface to ensure the effectiveness of pressure has to be regularly checked. Even if relevant preventive measures are implemented, the risk of DVT will be greatly reduced, but it cannot be completely avoided. Therefore, clinical doctors need to take individualized health education according to the different conditions of patients with gynecological malignant tumors to improve their quality of life and treatment compliance, and also to reduce the incidence of venous thrombosis.

## Discussion

Deep venous thrombosis is a frequently occurring disease with multiple factors and polygenic defects. It is a disease with high mortality. This study analyzed the risk factors of perioperative DVT patients with gynecological malignant tumors in China. It plays an important role in determining high-risk groups of DVT, implementing individualized prevention strategies, and reducing incidence rate and mortality. The clinical manifestations of DVT patients are non-specific, and this makes clinicians ignore the severity of their condition. This study shows that the most common clinical manifestations of DVT are swelling and pain of affected limbs, up to 68.23%. It is pointed out in the literature at home and abroad that the peripheral diameter of the lower leg should be measured when diagnosing DVT. Nicklas et al. (3) even proposed that the difference in the circumference of both legs I > 2 cm can be used as a clinical sign of DVT, which has high diagnostic sensitivity. Therefore, it is suggested that measuring the leg circumference in clinical work is also an inspection method that cannot be ignored.

In addition, the results of this study showed that when DVT had pulmonary symptoms, the main manifestations were dyspnea, chest pain, cough, and rapid heart rate. However, patients with pulmonary embolism are often complicated with some respiratory and cardiovascular diseases, and the symptoms and signs are lack of specificity. So it is difficult to distinguish the real cause of the patient and easy to miss diagnosis or misdiagnose. At present, clinical practice has confirmed that only 20% of patients with the traditional triple syndrome of pulmonary embolism (dyspnea, chest pain, and hemoptysis) exist at the same time. In addition, more patients with DVT and PE are asymptomatic, or their clinical symptoms and signs lack specificity, which makes the diagnosis difficult. Therefore, we should pay attention to risk factors and combine correct auxiliary examinations to diagnose DVT.

### Risk Factors of DVT

Previous studies have found that the three elements of the pathogenesis of DVT are slow blood flow, abnormal blood composition, and venous endothelial injury. Therefore, in the perioperative period of gynecological malignant tumors, any etiology causing these three factors can lead to DVT, including primary and secondary. In addition to age, which has been proved to be an independent and important risk factor for DVT, there are also many risk factors. This study found that among the risk factors of DVT, age >40 years old ranked first, suggesting that the incidence of DVT after 40 years old increased. This may be associated with increasing age. Rough vascular intima, increased chance of endothelial damage, increased production of procoagulant substances, and hypercoagulability are also closely related to the occurrence of DVT. Especially in patients with hypertension, hyperlipidemia, diabetes, coronary heart disease, cancer, etc. In addition, it may also be related to the increase of blood viscosity and the change of gonadotropin sex hormone. The results of this study show that the clear risk factors that can be found in patients with DVT also include surgery, smoking, heart disease, malignant tumor, hypertension, trauma, bed rest, previous DVT history, etc., and some patients have more than two risk factors, suggesting that multiple risk factors are synergistic and superimposed, which is more likely to lead to thrombosis.

The results of this study showed that the DVT related to surgery accounted for 27.51%. Preoperative fasting, anesthesia, long-term postoperative braking, and surgical trauma can affect limb movement, resulting in slow venous blood flow, and the damage of vascular wall can promote the adhesion and aggregation of platelets. Trauma, blood loss, and hypoxia can activate the coagulation system as stressors, which is conducive to thrombosis. Smoking was also a common risk factor in this study (18.56%). A large number of studies have shown that smoking is related to a variety of thrombotic diseases. Smoking can increase platelet activity and enhance platelet aggregation, where the level of fibrinogen in smokers increases. At the same time, carbon monoxide and nicotine produced by smoking can directly damage vascular endothelium and affect the antithrombotic effect of endothelium. The risk factors of heart disease include congenital heart disease, angina pectoris, and myocardial infarction, and can promote the occurrence of DVT. Studies have shown that the activation of platelet and coagulation system and fibrin renewal are significantly accelerated, coagulation factors are increased, and anticoagulants are reduced. Patients with cardiac insufficiency are blocked by venous reflux, causing blood stasis and DVT.

The relationship between malignant tumor and thrombosis is close and complex. DVT is not only a common complication of tumor patients, but also a sign and signal of occult tumor, which can provide opportunities for early diagnosis and treatment of tumor patients. Tumor can not only secrete procoagulant substances to promote platelet aggregation and release, but also secrete fibrinolytic activity inhibitors, resulting in hypercoagulable state. Some chemotherapeutic drugs can cause the deficiency of protein C and protein S and the decrease of antithrombin. In addition, tumor compression on blood vessels, long-term bed rest, and other factors can promote the formation of DVT. A high proportion of patients included in this study were found to have tumors, which once again confirmed the obvious correlation between tumors and DVT.

Long-term bed rest is the most important risk factor for DVT. Some studies have pointed out that the detection rate of DVT is only 15% in those who stay in bed for <1 week, and higher than 80% in those who stay in bed for more than 1 week. Due to the loss of the function of muscle pump, local blood flow stagnates in bedridden patients, causing venous dilatation, vascular endothelial injury, and coagulation factor accumulation activating the coagulation system and promoting the formation of thrombosis. The results of this study showed that 6.59% of DVT patients had bedridden factors. The previous history of DVT is one of the common risk factors for DVT recurrence, especially when these patients are in the period of major surgery or serious medical diseases requiring hospitalization. According to literature reports, the risk of DVT in hospitalized patients with a previous history of PTE or DVT was 7.9 times that of general hospitalized patients. In the 6-month course of DVT anticoagulation, the recurrence rate of DVT was twice that of patients without previous history of DVT. Diabetes and hypertension can lead to vascular endothelial cell injury, platelet activation, coagulation, fibrinolytic system imbalance, hypercoagulability state, thereby inducing the formation of DVT. Infection and rheumatic diseases can cause vascular endothelial injury, which creates favorable conditions for thrombosis. Polycythemia vera and primary thrombocytosis in hematological diseases can cause the blood to be in a state of high viscosity, resulting in slow blood flow and thrombosis.

In addition to the acquired risk factors, DVT also has genetic risk factors, including antithrombin deficiency, protein C, and protein S genetic deficiency. In recent years, it has been reported that the genetic risk factors of DVT are mainly coagulation factor V mutation and activated protein C resistance. Wang's study shows that FV Leiden mutation exists in the normal population of China. The relationship between FV Leiden, anti-APC, and venous thrombosis in China needs further study in the future. This study summarizes the characteristics of DVT patients in China, which is of great significance to understand and control venous thrombosis. It is an important strategy to be alert to risk factors, recognize the characteristics of clinical signs, and use accurate examination methods to diagnose DVT. Early correction of risk factors and early intervention and treatment are key factors to prevent DVT and reduce the mortality of patients.

### Clinical Implications of the Study

This article summarizes the current prevention and management of DVT in postoperative patients with gynecological malignant tumors, which provides evidence basis for clinical nurses and nursing managers. In the process of evidence application, we need to fully consider the specific situation of departments and the intention of patients. Most of the literatures included in this study are in foreign languages. Due to different regions, values, and medical levels, clinical nurses must be able to use the same principle according to the feasibility, suitability, clinical significance, and effectiveness of the evidence (26).

## Conclusion

Before the application of evidence, it is verified in practice according to the applicability and popularization of evidence. We should comprehensively evaluate patients' self-care ability, understanding ability, and coagulation function and then formulate personalized DVT prevention and management measures to improve patients' compliance, and finally effectively apply the best evidence to clinical practice.

## Data Availability Statement

The original contributions presented in the study are included in the article/supplementary material, further inquiries can be directed to the corresponding author.

## Author Contributions

JH and JT designed the study and prepared the manuscript. YG and JM collected the data. XD and SF analyzed the data. All authors read and approved the final manuscript.

## Conflict of Interest

The authors declare that the research was conducted in the absence of any commercial or financial relationships that could be construed as a potential conflict of interest.

## Publisher's Note

All claims expressed in this article are solely those of the authors and do not necessarily represent those of their affiliated organizations, or those of the publisher, the editors and the reviewers. Any product that may be evaluated in this article, or claim that may be made by its manufacturer, is not guaranteed or endorsed by the publisher.

## References

[1] Sung H Ferlay J Siegel RL Laversanne M Soerjomataram I Jemal A . Global cancer statistics 2020: GLOBOCAN estimates of incidence and mortality worldwide for 36 cancers in 185 countries. CA Cancer J Clin. (2021) 71:209–49. 10.3322/caac.21660 33538338

[2] Mohammed BM Cheng Q Ivanov IS Gailani D. Murine models in the evaluation of heparan sulfate-based anticoagulants. Methods Mol Biol. (2022) 2303:789–805. 10.1007/978-1-0716-1398-6_59 34626423 PMC8552346

[3] Nicklas JM Gordon AE Henke PK. Resolution of deep venous thrombosis: proposed immune paradigms. Int J Mol Sci. (2020) 21:2080. 10.3390/ijms21062080 32197363 PMC7139924

[4] Aydin E Bademci MS Kocaaslan C Oztekin A Denli YE. Iliofemoral deep venous thrombosis treatment modalities. J Vasc Surg Venous Lymphat Disord. (2020) 8:906. 10.1016/j.jvsv.2019.10.027 32800263

[5] Aufwerber S Heijne A Edman G Gravare SK Ackermann PW. Early mobilization does not reduce the risk of deep venous thrombosis after Achilles tendon rupture: a randomized controlled trial. Knee Surg Sports Traumatol Arthrosc. (2020) 28:312–9. 10.1007/s00167-019-05767-x 31679069 PMC6971132

[6] Komatsu H Shimada M Osaku D Deura I Sato S Oishi T . Deep vein thrombosis and serum D-dimer after pelvic lymphadenectomy in gynecological cancer. Int J Gynecol Cancer. (2020) 30:860–4. 10.1136/ijgc-2019-000914 32276932

[7] Dicenso A Bayley L Haynes RB. Accessing pre-appraised evidence: fine-tuning the 5S model into a 6S model. Evid Based Nurs. (2009) 12:99–101. 10.1136/ebn.12.4.99-b 19779069

[8] Zeng X Zhang Y Kwong JS Zhang C Li S Sun F . The methodological quality assessment tools for preclinical and clinical studies, systematic review and meta-analysis, and clinical practice guideline: a systematic review. J Evid Based Med. (2015) 8:2–10. 10.1111/jebm.12141 25594108

[9] Aromataris E Fernandez R Godfrey CM Holly C Khalil H Tungpunkom P. Summarizing systematic reviews: methodological development, conduct and reporting of an umbrella review approach. Int J Evid Based Healthc. (2015) 13:132–40. 10.1097/XEB.0000000000000055 26360830

[10] Borges MC Stein C Colpani V Barker TH Munn Z Falavigna M. How are systematic reviews of prevalence conducted? A methodological study. BMC Med Res Methodol. (2020) 20:96. 10.1186/s12874-020-00975-3 32336279 PMC7184711

[11] Buccheri RK Sharifi C. Critical appraisal tools and reporting guidelines for evidence-based practice. Worldviews Evid Based Nurs. (2017) 14:463–72. 10.1111/wvn.12258 28898556

[12] Farge D Frere C Connors JM Ay C Khorana AA Munoz A . 2019 International clinical practice guidelines for the treatment and prophylaxis of venous thromboembolism in patients with cancer. Lancet Oncol. (2019) 20:e566–81. 10.1016/S1470-2045(19)30336-5 31492632

[13] Liew NC Alemany GV Angchaisuksiri P Bang SM Choi G DE Silva DA . Asian venous thromboembolism guidelines: updated recommendations for the prevention of venous thromboembolism. Int Angiol. (2017) 36:1–20. 10.23736/S0392-9590.16.03765-2 27606807

[14] Wade R Sideris E Paton F Rice S Palmer S Fox D . Graduated compression stockings for the prevention of deep-vein thrombosis in postoperative surgical patients: a systematic review and economic model with a value of information analysis. Health Technol Assess. (2015) 19:1–220. 10.3310/hta19980 26613365 PMC4781245

[15] Venous Thromboembolism in Over 16s: Reducing the Risk of Hospital-Acquired Deep Vein Thrombosis or Pulmonary Embolism. London, National Institute for Health and Care Excellence (NICE) (2019). 32924386

[16] Streiff MB Holmstrom B Angelini D Ashrani A Bockenstedt PL Chesney C . NCCN guidelines insights: cancer-associated venous thromboembolic disease, version 2.2018. J Natl Compr Canc Netw. (2018) 16:1289–303. 10.6004/jnccn.2018.0084 30442731

[17] Wu L Cheng B. Analysis of perioperative risk factors for deep vein thrombosis in patients with femoral and pelvic fractures. J Orthop Surg Res. (2020) 15:597. 10.1186/s13018-020-02131-5 33302974 PMC7731763

[18] Guo X Zhang F Wu Y Gao L Wang Q Wang Z . Coagulation alteration and deep vein thrombosis in brain tumor patients during the perioperative period. World Neurosurg. (2018) 114:e982–91. 10.1016/j.wneu.2018.03.128 29588239

[19] Insin P Vitoopinyoparb K Thadanipon K Charakorn C Attia J McKay GJ . Prevention of venous thromboembolism in gynecological cancer patients undergoing major abdominopelvic surgery: a systematic review and network meta-analysis. Gynecol Oncol. (2021) 161:304–13. 10.1016/j.ygyno.2021.01.027 33563489

[20] Hajibandeh S Hajibandeh S Antoniou GA Scurr JR Torella F. Neuromuscular electrical stimulation for the prevention of venous thromboembolism. Cochrane Database Syst Rev. (2017) 11:D11764. 10.1002/14651858.CD011764.pub2 29161465 PMC6486105

[21] Pavon JM Adam SS Razouki ZA McDuffie JR Lachiewicz PF Kosinski AS . Effectiveness of intermittent pneumatic compression devices for venous thromboembolism prophylaxis in high-risk surgical patients: a systematic review. J Arthroplasty. (2016) 31:524–32. 10.1016/j.arth.2015.09.043 26525487

[22] Buesing KL Mullapudi B Flowers KA. Deep venous thrombosis and venous thromboembolism prophylaxis. Surg Clin North Am. (2015) 95:285–300. 10.1016/j.suc.2014.11.005 25814107

[23] Lieberman JR. Deep vein thrombosis prophylaxis: state of the art. J Arthroplasty. (2018) 33:3107–8. 10.1016/j.arth.2018.01.051 29573919

[24] Fan W Qiao T You Y Zhang J Gao J. Perioperative prevalence of deep vein thrombosis in patients with percutaneous kyphoplasty: a retrospective study with routine ultrasonography. Medicine. (2020) 99:e19402. 10.1097/MD.0000000000019402 32150087 PMC7478572

[25] Li J Zhu Y Chen W Zhao K Zhang J Meng H . Incidence and locations of deep venous thrombosis of the lower extremity following surgeries of tibial plateau fractures: a prospective cohort study. J Orthop Surg Res. (2020) 15:605. 10.1186/s13018-020-02136-0 33317585 PMC7735415

[26] Matsusaki T Sakai T Boucek CD Abu-Elmagd K Martin LM Amesur N . Central venous thrombosis and perioperative vascular access in adult intestinal transplantation. Br J Anaesth. (2012) 108:776–83. 10.1093/bja/aes016 22362673

[27] Gantz O Mulles S Zagadailov P Merchant AM. Incidence and cost of deep vein thrombosis in emergency general surgery over 15 years. J Surg Res. (2020) 252:125–32. 10.1016/j.jss.2020.03.022 32278966

[28] Kahn SR Morrison DR Diendere G Piche A Filion KB Klil-Drori AJ . Interventions for implementation of thromboprophylaxis in hospitalized patients at risk for venous thromboembolism. Cochrane Database Syst Rev. (2018) 4:D8201. 10.1002/14651858.CD008201.pub3 29687454 PMC6747554

[29] Zhao W McArthur A Yu Z Hu Y Luo J. Prevention of venous thromboembolism in postoperative abdominal patients: a best practice implementation project. JBI Database System Rev Implement Rep. (2018) 16:1887–901. 10.11124/JBISRIR-2017-003665 30204673

[30] Sheth T Natarajan MK Hsieh V Valettas N Rokoss M Mehta S . Incidence of thrombosis in perioperative and non-operative myocardial infarction. Br J Anaesth. (2018) 120:725–33. 10.1016/j.bja.2017.11.063 29576113

